# Year‐round weather alters nest‐provisioning rates in a migratory owl

**DOI:** 10.1002/ece3.10333

**Published:** 2023-07-23

**Authors:** Eliza D. Stein, Stephen R. Midway, Brian D. Linkhart

**Affiliations:** ^1^ School of Renewable Natural Resources Louisiana State University and Agricultural Center Baton Rouge Louisiana USA; ^2^ Department of Oceanography and Coastal Sciences Louisiana State University Baton Rouge Louisiana USA; ^3^ Department of Organismal Biology and Ecology Colorado College Colorado Springs Colorado USA

**Keywords:** climate change, conservation, Flammulated Owl, nest provisioning, precipitation, temperature

## Abstract

As global temperatures and precipitation become more extreme, habitat specialists are at particular risk of being pushed past their environmental tolerance limits. Flammulated Owls (*Psiloscops flammeolus*) are small migratory owls that breed in temperate conifer forests of western North America. Their highly specialized nesting and foraging requirements make them indicators of ecosystem health. Using 17 years of nest observations, we investigated how annual weather patterns affected Flammulated Owl nesting and foraging behaviors during the breeding season. We used generalized linear models with a changepoint parameter to evaluate nest provisioning and nestling growth rates in years of extreme temperature and precipitation. We also evaluated how adult mass, division of labor, and productivity varied based on precipitation and temperature. Compared to wet and warm years, adults made more frequent prey deliveries to nestlings in dry and cold years, particularly early in the night and early in the season, and they experienced earlier changepoints in these years. We found a significant effect of temperature on the number of fledglings in broods, but weather did not affect other variables including productivity, nestling growth rates, adult masses, and division of labor. Our findings suggest that extreme annual weather patterns influence insect prey availability during the Flammulated Owl breeding season, forcing adults to work harder to provision for nests during dry and cold years. While productivity and nestling growth did not vary between years, these may incur a long‐term tradeoff in adult survival.

## Graphical abstract

Using 17 years of nest observations, we investigated how annual weather patterns affected Flammulated Owl nesting and foraging behaviors during the breeding season. Nest‐provisioning rates were higher in dry and cold years compared to wet and warm years, but the weather did not affect nestling growth rates, adult mass, division of labor, or nest productivity. These findings suggest that Flammulated Owls have accommodated changing weather patterns by adjusting their nest‐provisioning strategies, requiring adults to work harder in cold and dry years.

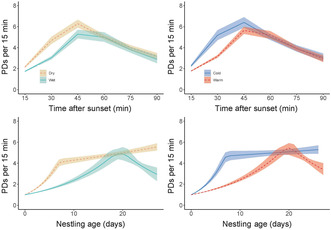

## 1 INTRODUCTION

Anthropogenic climate change increases the intensity of weather fluctuations in temperate ecosystems, pushing many organisms past their tolerance limits (Addo‐Bediako et al., 2000; Deutsch et al., 2008). Habitat specialists are particularly vulnerable to these fluctuations and are often the first species in which evidence of ecosystem stress is detected (Colles et al., 2009; Julliard et al., 2004; McKinney, 1997). Long‐term studies of habitat specialists that elucidate changes in these organisms' behaviors can, therefore, provide valuable insights into the consequences of climate change for the overall ecosystem (Clavel et al., 2011; Donovan et al., 2002).

Flammulated Owls (*Psiloscops flammeolus*) have unique breeding requirements and life histories, making them indicators of environmental change in their forest ecosystems (Linkhart & Reynolds, 1997; McCallum, 1994; Van Woudenberg, 1999). Across their breeding range in western North America, these Neotropical migrants (Linkhart et al., 2016) are designated as a sensitive species by the United States Forest Service (McCallum, 1994) and a species of conservation concern by the United States Fish and Wildlife Service (USFWS) (2021), and species of special concern in Canada (COSEWIC, 2001). Flammulated Owls exhibit a life history strategy similar to large raptors by having high adult survival and a low annual reproductive rate (mean clutch size is 2.5 ± 0.1 eggs) with no evidence of renesting (Linkhart & Reynolds, 2006, 2007). Flammulated Owls are insectivorous and are most often associated with older forests containing yellow pine (subfamily Ponderosae) in the Rocky Mountains (breeding), Sierra Nevada Mountains (breeding), and Sierra Madre Mountains (breeding, resident, and wintering), where they primarily capture and feed on Lepidoptera in tree crowns and Coleoptera and Orthoptera on the ground (Marshall, 1957; Reynolds & Linkhart, 1992; Ross, 1969). Despite their reliance on small‐bodied prey, Flammulated Owls are single‐prey loaders, meaning they only carry one item of prey at a time. This allows for the number of prey items delivered to nests to be directly quantified by the number of visits an adult makes to the nest. It is also more energetically demanding for single‐prey loaders (as opposed to multiple‐prey loaders) to increase the number of prey they bring to the nest because they must make more foraging trips, rather than increase the number of prey captured per trip (Lessells & Stephens, 1983). Adult Flammulated Owls exhibit a strong division of labor, with females assuming all nest‐caretaking roles and males acting as the principal foragers (Linkhart & McCallum, 2020).

Seasonal weather patterns have profound effects on plant and insect communities, and thus on the breeding birds that rely on them (Barnett & Facey, 2016; Desante & Saracco, 2021; Ladwig et al., 2016). While several studies have examined the impacts of breeding season precipitation and air temperature on bird energetics (Haftorn & Reinertsen, 1985; Ortega‐Jiménez et al., 2010; Schifferli et al., 2014), nest provisioning and division of labor (Barras et al., 2021; Low et al., 2008; Radford et al., 2001), nestling growth (Imlay et al., 2017; Kruuk et al., 2015; Pérez et al., 2016), productivity (Demay & Walters, 2019; Fisher et al., 2015; Gullett et al., 2015), and insect abundance (Cucco & Malacarne, 1996; Grüebler et al., 2008), few studies considered the effects of precipitation and temperature outside of the breeding season on the breeding ecology of insectivorous birds (Desante & Saracco, 2021). In the temperate forests of the southwestern United States, year‐round precipitation plays an important role in determining plant communities and overall forest health (Arizpe et al., 2020; Kaufmann et al., 2007; Sheppard et al., 2002; Truettner et al., 2018). Fall, winter, and spring temperatures also mediate diapause in insects that reach peak abundances in summer (Bale et al., 2002; Kevan & Kendall, 1997), and the length and severity of winter freezes influence survival of many other insects (Bale & Hayward, 2010; Irwin & Lee, 2000; Stockton & Loeb, 2021). Thus, in cold and dry years, insectivores may exhibit prey‐switching behavior that favors an increased quantity of small, abundant prey over harder to locate large, less abundant prey (Cauchard et al., 2021; Mägi et al., 2009).

We investigated the effects of annual precipitation and ambient air temperature (hereafter, temperature) on Flammulated Owl nest‐provisioning rates, division of labor, body condition, nestling growth, and productivity using long‐term nest observation data. Specifically, we used changepoint models to test how annual precipitation and temperature affect the rate of prey deliveries and division of labor (1) throughout the night and (2) over the course of the nestling period. We predicted that nest‐provisioning rates would be higher in dry years than in wet years, and higher in cold years than in warm years, due to a lower abundance of high‐quality prey, which would necessitate more frequent deliveries of the more relatively abundant low‐quality prey. In cold years, prey delivery rates might also be higher because of increased nestling energy demands for maintaining homeothermy. We anticipated that prey delivery rates would drop off later in the night and later in the season in dry and cold years because of the increased foraging time required to meet nestling energy demands. We also predicted that adult females would increase their contributions to nest provisioning in dry and cold years to compensate for reduced male efficiency and higher nestling energy needs, which could lead to poor female body condition. Finally, we tested for an effect of precipitation and temperature on clutch size, brood size, the number of fledglings from each nest, and nestling growth rates. We predicted that both dry and cold years would result in lower overall productivity and slower nestling growth than wet and warm years due to the challenges adults face in meeting nestling, and their own, energy requirements.

## 2 METHODS

### 2.1 Study system

Nest observations for this study took place at the Manitou Experimental Forest (39.1, −105.1), a 6758‐ha tract within the Pike National Forest in central Colorado, USA. Ridgetops (maximum elevation = 2800 m) and south‐ and west‐facing slopes consist of open stands of old‐growth ponderosa pine (*Pinus ponderosa*) mixed with Douglas fir (*Pseudotsuga menziesii*) and limber pine (*P. flexilis*), with north‐ and east‐facing slopes consisting of dense stands of Douglas fir and blue spruce (*Picea pungens*); quaking aspen (*Populus tremuloides*) and blue spruce dominate drainage bottoms (minimum elevation = 2500 m). Mean monthly precipitation is 67.9 mm in July and 5.1 mm in January, with 46% of annual precipitation occurring during the Flammulated Owl breeding season (May–July; Frank et al., 2021). Average temperatures are 17.0°C in July and −4.0°C in January, with snow often covering the ground from December through February (Frank et al., 2021; Ortega et al., 2014).

### 2.2 Nest observation

We located all nests in the study area annually from 2004 to 2020 using methods developed by BDL (BDL, unpublished data; Linkhart & Reynolds, 2007; Reynolds & Linkhart, 1984). Briefly, we checked all tree cavities (excavated by picid woodpeckers) with diameters >4 cm using cameras mounted on telescopic poles. Cavities were usually checked weekly, beginning at the onset of laying (late May) until fledging (mid‐July). We estimated nestling age by (1) backdating from the date of fledging (mean duration of nestling period was 23 days; Reynolds & Linkhart, 1987), (2) comparing camera photos showing plumage and morphometric development to photos of known‐age nestlings, and/or (3) comparing lengths of primary feathers to primary lengths from known‐age nestlings and fledglings (BDL, unpublished data).

To measure the rate of prey deliveries throughout the nestling period, we observed nests for at least one 15‐min interval per week and recorded the total number of visits made to the nest by the attendant male or female. We varied the time of night to ensure a balance of early‐ and late‐evening observations for each nest, and we recorded rates as the number of deliveries (as counts) per 15 min. Following methods developed by Reynolds and Linkhart (1987), a prey delivery was attributed to the male if any one of the following criteria was met: (1) the female was known to be on the nest, (2) male vocalizations accompanied the delivery, or (3) the female was heard vocalizing off‐nest while a second owl entered the cavity. A prey delivery was attributed to the female if any one of the opposing conditions was met, and deliveries made by an adult of unknown sex were not included in analysis. We omitted any intervals when nests were not observed for the full 15 min, and nest observations that occurred later than 90 min after sunset were not included in the analysis due to small sample sizes. Nonetheless, because prey delivery rates throughout the entire Flammulated Owl nestling period are maintained at their highest levels during this 90‐min period (40%–80% higher than the rest of the night; Reynolds & Linkhart, 1987), we believe this window is most insightful, and sufficient, for detecting meaningful patterns on individual nights and over the full nestling period. We did not evaluate prey delivery rates in 2008, 2010, or 2016.

To estimate nest productivity, we recorded the clutch or brood size at each nest check. We estimated the number of fledglings by directly observing nestlings fledge from their nest, locating the fledglings after they had fledged, or observing nestlings within 1–3 days prior to their anticipated fledging. Brood sizes that were smaller than original clutch sizes were judged to be the result of unhatched eggs, partial or full nest predation during the incubation stage, or nest abandonment. Instances where the number of fledglings was smaller than brood size reflected partial or full predation during the nestling stage, siblicide, starvation, or nest abandonment. Nests with unknown fates were excluded from all analyses, but observations of failed nests were included if the observations preceded nest failure.

### 2.3 Mass

We captured adult Flammulated Owls at nests or in a mist net with playback at least once, and in some cases multiple times, each breeding season. Individuals were massed (g) during each capture using a Pesola™ spring‐loaded scale (accurate to 0.5 g). Multiple masses of the same adult within a season were uncommon but were recorded as separate observations if they occurred on different days, as were masses of the same individual in different years. We included all adult masses in our analysis, even if the nest fate was unknown.

To weigh nestlings, we ascended nest trees during the day with a ladder or, if the tree was live, climbing hooks. All nestlings in a brood were removed from the nest and massed with a digital scale at least twice. Whenever possible, we captured owlets after observing them fledge and recorded their masses as the final day of the nestling period.

### 2.4 Meteorological data

We downloaded hourly precipitation and temperature data for the Manitou Experimental Forest between 2004 and 2020 from the online Forest Service Research Data Archive (Frank et al., 2021). Monthly meteorological data from 1950 to 2000 was accessed through the National Weather Service historical data portal for Colorado Springs (NOAA, 2022).

*Precipitation* was calculated by totaling the precipitation for the entire year preceding the breeding season, beginning on 1 June of the previous year and ending on 31 May of the breeding year. Historical precipitation was totaled using the same start and end dates for each year from 1950 to 2000. Only 2 years of our study fell above the historical mean for total annual precipitation, and the mean annual precipitation from 2004 to 2020 (300.3 mm) was 71.8% of the mean annual precipitation from 1950 to 2000 (418.1 mm). We therefore, classified years falling above 71.8% of the historical mean as *wet*, and years falling below 71.8% were classified as *dry* (no years were equal to 71.8% of the historic mean).

Similarly, *temperature* was calculated by computing the mean daily minimum temperature between 1 June and 31 May for the historical period (1950–2000) and for our nest observation period. No years in our study fell within the 100% quartile of historical minimum temperatures, and the annual mean minimum temperature from 2004 to 2020 (−3.0°C) was 46.5% higher than the annual mean minimum temperature from 1950 to 2000 (−6.4°C). We therefore classified years between 2004 and 2020 as *warm* or *cold* based on whether they fell above or below 46.5% of the historical mean.

### 2.5 Statistical analysis

Before considering *precipitation* and *temperature* as separate predictors, we tested for correlation between total yearly precipitation and average daily minimum temperatures using a Pearson's paired sample correlation test in the R stats package (R Core Team, 2021). *Precipitation* and *temperature* were positively correlated (Pearson's correlation coefficient = .67, *p* < .001); we constructed one group of models for *precipitation* and a separate group for *temperature*.

Initially, we included *precipitation* and *temperature* as continuous numerical variables in all models. However, in addition to not seeing any initial effect of *temperature* and *precipitation* when treated as continuous, we were concerned that effects could be nonlinear due to extreme values. Consequently, we categorized the data into groups above and below the stated percentages of the historical means.

To determine how and when air temperature exerted the strongest effects on prey delivery rates, we ran Poisson GLMs using maximum likelihood estimation that tested for effects of three measures of air temperature during two different time periods. We considered (1) average daily air temperature, (2) minimum daily air temperature, and (3) maximum daily air temperature from (1) June–July (encompassing the nestling period) and (2) June–May (the 12 months preceding the initiation of breeding). We ultimately selected June–May average minimum daily air temperature, the model with the lowest Akaike information criterion (AIC) value, for subsequent analyses (Table A1 in Appendix 1).

To determine which time period to include in precipitation models, we considered total precipitation from (1) June–July (encompassing the nestling period), (2) June–May (the 12 months preceding the initiation of breeding), (3) January–June (capturing the major spring precipitation pulse), and (4) July–December (capturing the major summer precipitation pulse). The model using June–May precipitation as the predictor had the lowest AIC value and was, therefore, used for subsequent precipitation analyses.

To test for effects of *precipitation* and *temperature* on prey delivery rates, we used Poisson GLMs with a changepoint parameter. Estimating changepoints was important because previous studies showed that Flammulated Owl prey delivery rates were nonlinear through time. Specifically, prey delivery rates increased for a short period immediately after sunset before dropping off later in the night; similarly, prey deliveries initially increased throughout the nestling stage before decreasing in the days preceding fledging (Reynolds & Linkhart, 1987). We adopted a Poisson‐distributed Bayesian changepoint model to infer joined regression models for individual segments of a dataset using the R package mcp (Lindeløv, 2020). Our final model formulation contained one changepoint and two segments, the first segment representing the early‐evening or early‐nestling stage, and the second segment representing the late‐evening or late‐nestling stage. The response variable for this logit‐link model was the prey delivery rate, treated as count data (number of deliveries per 15 min); the predictor variable was time after sunset (min) for nightly models and nest age (days) for seasonal models. We used uninformative priors to estimate changepoints (uniform distribution between the minimum and maximum values of *x* and variance of 1000) and slopes (normally distributed around a mean of 0 and a variance of 1000) and assigned an intercept of 0 to our models because prey deliveries do not take place before sunset or before hatching. Each model was run separately for wet years, dry years, warm years, and cold years, with three chains with 20,000 iterations, burn‐in of 4000, and thinning of 1. Model convergence was evaluated using Pearson residuals (plotted against fitted values), trace plots (ensuring convergence of all three chains), and $\hat{R}$ values (<1.1). We evaluated the overlap of 95% credible intervals (CRIs) between variables to determine differences based on *precipitation* and *temperature*. If any of the 95% CRIs overlapped between models, we considered there to be no significant difference. The formula for these models was adapted from Lindeløv (2020):

(Segment 1) $$\log\left(\gamma_{i}\right)=\left[x_{i}>{∆}_{0}\right]\left[x_{i}<{∆}_{1}\right]\beta_{1}x_{1,i}+$$

(Segment 2) $$\left[x_{i}>{∆}_{1}\right]\beta_{2}x_{2,i}$$

where

$$y_{i}∼\text{Poisson}\left(g^{-1}\left(\gamma_{i}\right)\right)$$

$$x_{1,i}=\min\left\{x_{i},{∆}_{1}\right\}-{∆}_{0}$$

$$x_{2,i}=\min\left\{x_{i},{∆}_{2}\right\}-{∆}_{1}$$

Next, we evaluated whether *precipitation* and *temperature* influenced the extent of division of labor between males and females. We initially used a Bayesian changepoint model with a beta distribution for the response variable to test the nightly and seasonal effects of climate on the proportion of total prey deliveries made by the female, but the CRIs for the changepoints were so broad (95% CRI ranged from 17 to 61 min for a single night and from 1 to 23 days for the nestling period) that we instead adopted a model without changepoints. After exploratory analysis, we constructed a Bayesian GLM with a beta distribution for the response variable to model the proportion of total prey deliveries attributable to the female throughout (1) a single night and (2) the breeding season for wet, dry, warm, and cold years. We used the package R2jags (Su & Yajima, 2021) to work with the models in R but connected with JAGS (Plummer, 2022) for the MCMC sampling. We used uninformative priors for the slopes and intercepts (uniform distributions with a mean of 0 and variance of 1000). Models ran on three chains with 10,000 iterations, burn‐in of 1000, and thinning of 1. Like our changepoint model described above, model convergence was evaluated using residuals, trace plots, and $\hat{R}$ values (<1.1). We compared CRIs for wet and dry years and for warm and cold years; nonoverlapping 95% CRIs indicated significant differences in division of labor.

To assess the effects of *precipitation* and *temperature* on owlet growth, we used a Bayesian linear mixed effects model (Gaussian distribution) with a single changepoint, with *nestling age* as the fixed effect and *mass* as the response variable. To account for variation in growth rates across individuals, we included band number as a random effect (Cox et al., 2019). This allowed the changepoint and slopes to vary by nestling, and the hyperparameter estimates of the changepoint and slopes took into account the number of measurements for each nestling. For this changepoint model, we included an intercept parameter because owlets have a nonzero mass at hatching. We used uninformative priors to estimate changepoints (uniform distribution between the minimum and maximum values of *x* and variance of 1000), slopes (normally distributed around a mean of 0 and a variance of 1000), and intercepts (same priors as slopes). Models ran on three chains with 4000 iterations, burn‐in of 2000, and thinning of 1. Again, we ran the model for wet, dry, warm, and cold years and defined significance as nonoverlapping 95% CRIs between variables.

To test for an effect of climate on adult mass throughout the nestling period, we used a Bayesian linear model (Gaussian distribution) with *mass* as the response and *Julian day* as the predictor. This model was run separately for wet, dry, warm, and cold years. Analysis was also performed separately for males and females because of differences in nest‐provisioning rates, and because only females exhibited brooding behavior (Reynolds & Linkhart, 1987). To account for the nonindependence of measurements from the same individual, we included band number as a random effect (Cox et al., 2019). This allowed the intercepts and slopes to vary by band number, and the hyperparameter estimates of the intercepts and slopes took into account the number of measurements for each individual. We used uninformative priors for the slopes and intercepts (uniform distributions with a mean of 0 and variance of 1000). Models ran on three chains with 200,000 iterations, burn‐in of 50,000, and thinning of 1. To determine whether adult mass changed throughout time, we assessed whether the 95% CRI of the posterior estimate of the slope coefficients overlapped zero. CRIs that did not overlap zero indicated significant changes in mass over time; CRIs that did not overlap with each other indicated significant effects of *temperature* and *precipitation* on adult mass.

Finally, we tested for an effect of *precipitation* and *temperature* on nest productivity. We used a Poisson GLM (fit in JAGS) to compare mean clutch size, brood size, and number of fledglings between wet and dry years and between warm and cold years. We used uninformative priors for the slopes and intercepts (uniform distributions with a mean of 0 and variance of 1000). Models ran on three chains with 20,000 iterations, burn‐in of 4000, and thinning of 1. Any 95% CRIs that did not overlap zero indicated significant effects of *precipitation* and *temperature* on productivity. We also compared the posterior distributions of the mean clutch size, brood size, and number of fledglings between wet and dry years and between warm and cold years and evaluated whether the CRIs overlapped between predictor variables.

All analyses were performed in R version 4.3.0, and we used the tidyverse and ggdist packages for data cleanup and visualization (Kay, 2021; R Core Team, 2023; Wickham et al., 2019).

## 3 RESULTS

We determined prey delivery rates, nestling mass, and/or productivity at 400 unique owl nests between 2004 and 2020, consisting of 181 nests (45%) and 219 nests (55%) in wet versus dry years, respectively, and 221 nests (55%) and 179 nests (45%) in warm versus cold years (Table 1). Annual precipitation during the 17 years of nest observation ranged from 180.34 to 506.98 mm, with an average of 300.32 ± 81.67 mm (mean ± SD). Average daily minimum temperatures for each year ranged from −3.95 to −2.18°C, with a mean of −2.98 ± 0.48°C.

**TABLE 1:** Yearly summary of climate and nest productivity.

Year	Total monthly precipitation (mm)	Mean daily minimum temperature (°C)	Wet/dry	Warm/cold	Nest count (prey deliveries)	Nest count (productivity and mass)	Mean clutch size	Mean brood size	Mean fledglings
2004	224.03	−3.07	Dry	Cold	11	21	3.00	2.74	2.45
2005	326.64	−2.63	Wet	Warm	11	27	2.75	1.81	1.74
2006	180.34	−3.30	Dry	Cold	4	22	2.78	1.90	0.55
2007	455.68	−2.91	Wet	Warm	7	24	2.40	1.74	1.29
2008	286.77	−3.36	Dry	Cold	0	22	2.38	1.48	1.23
2009	276.61	−2.99	Dry	Cold	9	23	2.72	1.48	1.17
2010	333.25	−3.95	Wet	Cold	0	24	2.59	2.05	1.42
2011	340.11	−2.93	Wet	Warm	9	20	2.68	1.89	1.25
2012	308.61	−2.68	Wet	Warm	9	26	2.69	2.05	1.19
2013	236.73	−3.65	Dry	Cold	15	28	2.65	2.04	1.75
2014	279.15	−2.88	Dry	Warm	15	37	2.67	2.28	2.05
2015	506.98	−2.32	Wet	Warm	8	23	2.55	1.87	1.70
2016	303.02	−2.54	Wet	Warm	0	18	2.90	2.59	2.33
2017	291.08	−2.18	Dry	Warm	7	27	2.83	2.33	2.15
2018	302.77	−2.58	Wet	Warm	10	19	2.61	2.21	2.00
2019	219.71	−3.48	Dry	Cold	9	25	2.61	1.80	1.60
2020	233.93	−3.25	Dry	Cold	13	14	3.08	2.62	2.07

*Note*: *Wet*/*dry* and *warm*/*cold* values assigned based on whether the year's precipitation or temperature value fell above or below the historical mean from 1950 to 2000. Mean values are the arithmetic means of all nests observed each year.

### 3.1 Prey delivery rates

We recorded a total of 4704 prey deliveries at 137 unique nests, including 54 nests (39%) and 83 nests (61%) in wet versus dry years, respectively, and 76 nests (55%) and 61 nests (45%) in warm versus cold years.

Overall, prey delivery rates were higher in dry years than in wet years (Table 2). Throughout a single night, mean, maximum, and minimum rates were 17%, 24%, and 19% higher, respectively, in dry years than in wet years. Throughout the nestling period, mean prey delivery rates were 24% higher in dry years than in wet years, maximum rates were not significantly different, and minimum rates were identical (=1) for both dry and wet years.

**TABLE 2:** Mean, minimum, and maximums of posterior estimates and their 95% credible intervals (CRI) for the number of prey deliveries (PDs) given per 15 min.

	PDs per 15 min		
	Estimate	Lower 2.5%	Upper 97.5%
Time after sunset			
Mean			
Wet*	3.72*	**3.44**	**4.02**
Dry*	4.37*	**4.11**	**4.63**
Warm	3.89	3.67	4.13
Cold	4.46	4.12	4.81
Minimum			
Wet*	1.74*	**1.70**	**1.79**
Dry*	2.16*	**2.08**	**2.24**
Warm*	1.78*	**1.75**	**1.82**
Cold*	2.28*	**2.17**	**2.38**
Maximum			
Wet*	5.29*	**4.88**	**5.71**
Dry*	6.28*	**5.92**	**6.66**
Warm	5.65	5.31	5.98
Cold	6.40	5.92	6.90
Nestling age			
Mean			
Wet*	**3.54**	**3.19**	**3.95**
Dry*	**4.39**	**4.16**	**4.63**
Warm*	**3.75**	**3.45**	**4.09**
Cold*	**4.49**	**4.19**	**4.81**
Minimum			
Wet	1	1	1
Dry	1	1	1
Warm	1	1	1
Cold	1	1	1
Maximum			
Wet	4.89	4.39	5.52
Dry	5.89	5.41	6.39
Warm	5.44	4.83	5.92
Cold	5.47	4.90	6.08

*Note*: CRIs that do not overlap between *wet*/*dry* or *warm*/*cold* years (bold and marked with an asterisk) indicate a significant effect.

Prey delivery rates were slightly higher overall in cold years than in warm years, although we noted more overlap of CRIs (Table 2). Throughout a single night, mean and maximum rates were not significantly different, but minimum rates were 28% higher in dry years than in warm years. Throughout the nestling period, mean rates were 20% higher in dry years than in wet years, maximum rates were not significantly different, and minimums rates were identical (=1).

**FIGURE 1:**
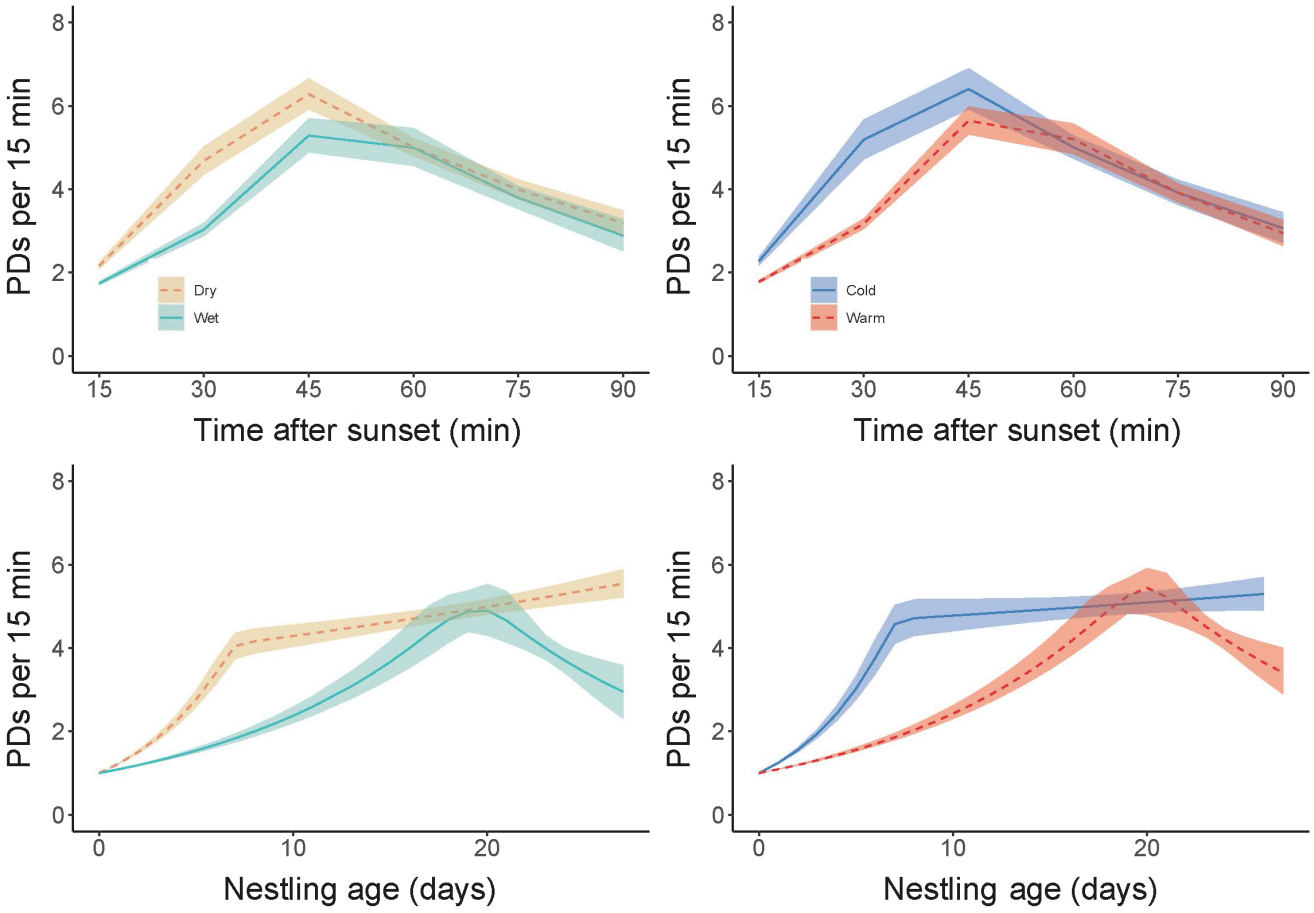
Prey delivery (PD) rates throughout the night (top) and throughout the nestling period (bottom) differ based on precipitation (left) and temperature (right). Solid and dashed lines represent the fitted value computed using the mean of posteriors predicted from Bayesian changepoint models, and shaded ribbons represent the 95% credible interval of posteriors. *Wet* = green/solid, *dry* = yellow/dashed, *warm* = red/dashed, *cold* = blue/solid.

**FIGURE A1:**
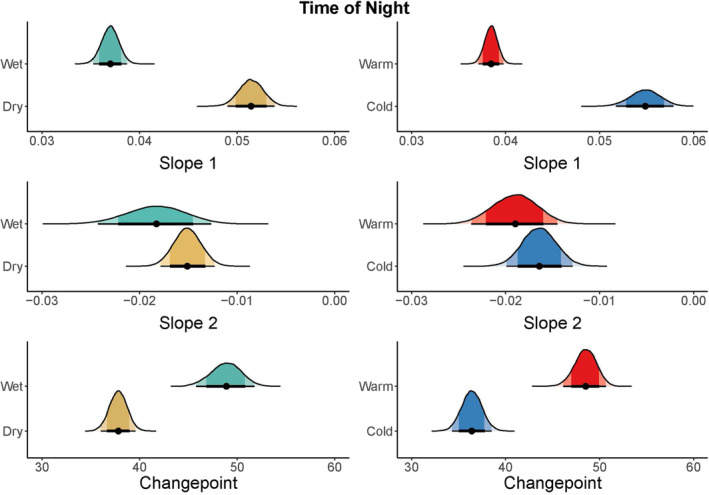
Distribution of posteriors predicted from Bayesian changepoint models of prey delivery rates throughout a single night. Black bars represent the mean (dot), 80% credible interval (thick bar), and 95% credible interval (thin bar). Darkest shades represent the 80% credible interval, medium shades represent the 95% credible interval, and lightest shades represent 100% of posterior estimates.

All prey delivery models estimated a single changepoint (Table 3; Figure 1). At the beginning of the night, deliveries increased at a higher rate in dry and cold years than in wet and warm years (Figure A1 in Appendix 1). At the end of the night, deliveries decreased at similar rates between dry and wet years and between cold and warm years. The changepoint occurred 29% earlier in the night in dry years and 33% earlier in cold years than in wet and warm years, respectively.

**TABLE 3:** Mean and 95% credible interval (CRI) for the posterior estimates of changepoints and slopes from prey delivery changepoint models.

	Posterior estimate		
	Mean	Lower 2.5%	Upper 97.5%
Time after sunset			
Changepoint			
Wet*	**48.87**	**45.85**	**51.82**
Dry*	**37.85**	**36.06**	**39.64**
**Warm**	**48.50**	**50.75**	**46.22**
Cold*	**36.41**	**38.53**	**34.32**
Slope 1			
Wet*	**0.04**	**0.04**	**0.04**
Dry*	**0.05**	**0.05**	**0.05**
Warm*	**0.04**	**0.04**	**0.04**
Cold*	**0.05**	**0.06**	**0.05**
Slope 2			
Wet	−0.02	−0.02	−0.01
Dry	−0.02	−0.02	−0.01
Warm	−0.02	−0.01	−0.02
Cold	−0.02	−0.01	−0.02
Nestling age			
Changepoint			
Wet*	**19.10**	**16.92**	**20.92**
Dry*	**6.95**	**6.32**	**7.52**
Warm*	**19.60**	**20.93**	**17.42**
Cold*	**6.99**	**7.68**	**6.34**
Slope 1			
Wet*	**0.09**	**0.08**	**0.10**
Dry*	**0.20**	**0.19**	**0.22**
Warm*	**0.09**	**0.10**	**0.082**
Cold*	**0.22**	**0.24**	**0.20**
Slope 2			
Wet*	**−0.08**	**−0.13**	**−0.03**
Dry*	**0.02**	**0.01**	**0.02**
Warm*	**−0.07**	**−0.03**	**−0.11**
Cold*	**0.01**	**0.01**	**0.00**

*Note*: CRIs that do not overlap between *wet*/*dry* or *warm*/*cold* years (bold and marked with an asterisk) indicate a significant effect.

**FIGURE A2:**
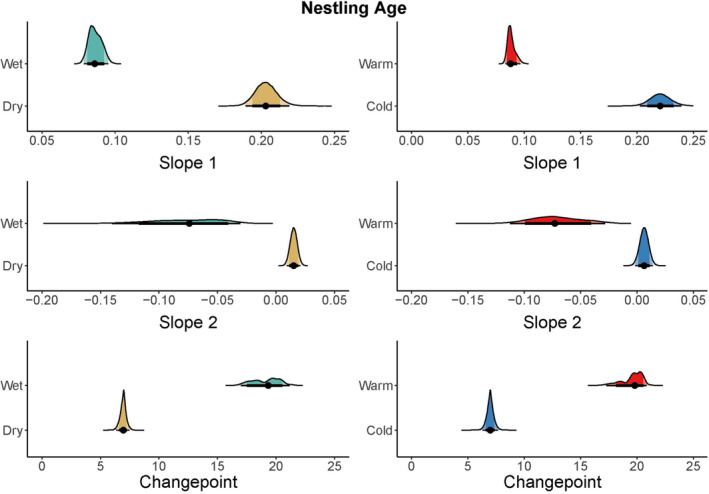
Distribution of posteriors predicted from Bayesian changepoint models of prey delivery rates throughout the nestling period. Black bars represent the mean (dot), 80% credible interval (thick bar), and 95% credible interval (thin bar). Darkest shades represent the 80% credible interval, medium shades represent the 95% credible interval, and lightest shades represent 100% of posterior estimates.

At the beginning of the nestling period, prey deliveries increased at a higher rate in dry and cold years than in wet and warm years (Table 3; Figure A2 in Appendix 1). At the end of the nestling period, after the changepoints, rates continued to increase in dry and cold years but began to decrease in wet and warm years. The changepoints occurred 75% earlier in dry years and 80% earlier in cold years than in wet and warm years, respectively.

### 3.2 Division of labor

Of 4078 total prey deliveries observed throughout the study, males accounted for more than three times as many as females (3090 vs. 988 deliveries, respectively).

**FIGURE 2:**
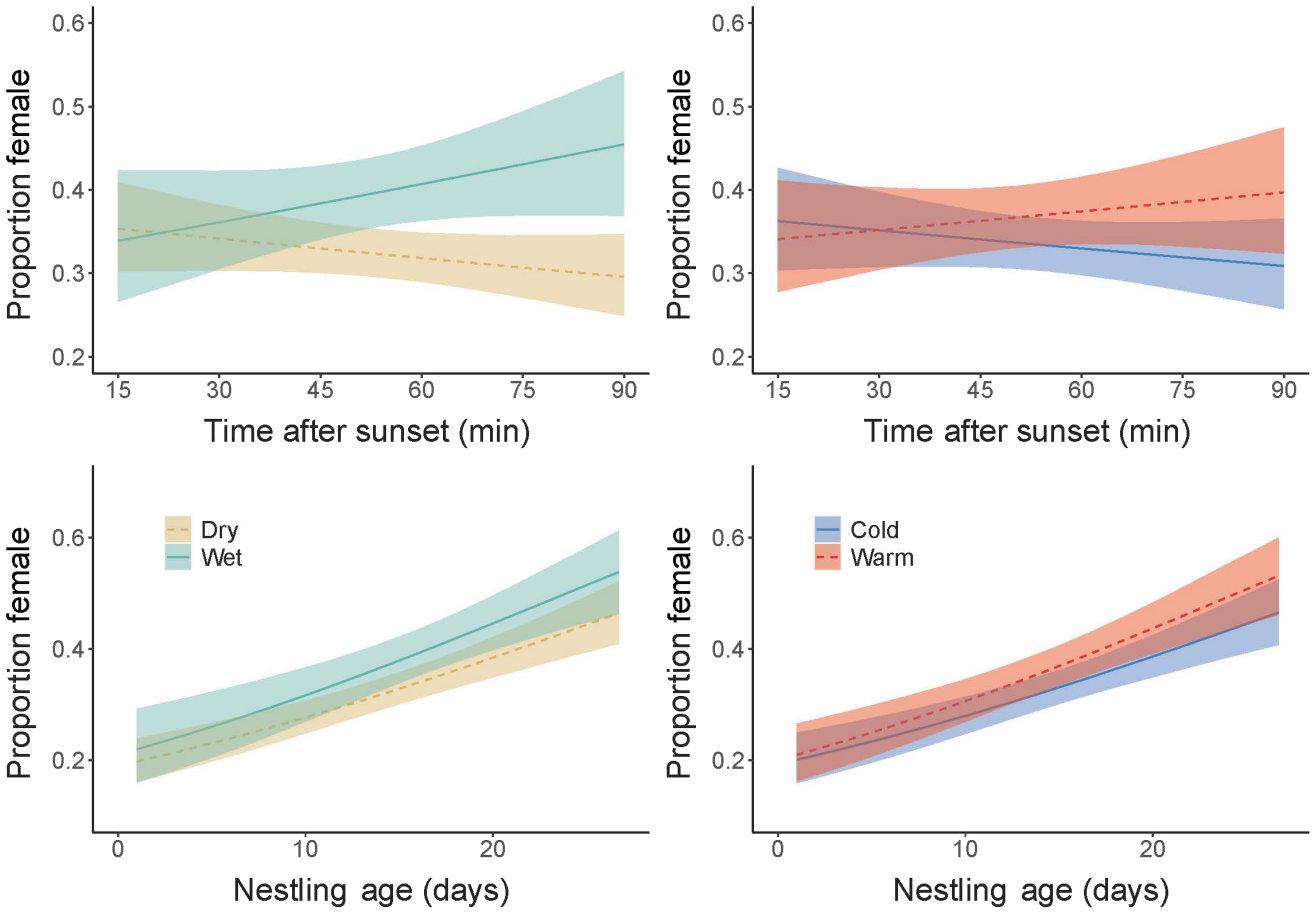
Bayesian linear model (beta distribution) of the proportion of prey deliveries given by the female throughout the night (top) and throughout the nestling period (bottom). Solid and dashed lines represent the fitted value computed using the mean of posteriors, and shaded ribbons represent the 95% credible interval of posteriors. *Wet* = green/solid, *dry* = yellow/dashed, *warm* = red/dashed, *cold* = blue/solid.

Across all weather groups, the proportion of male prey deliveries remained constant over the course of a single night (Figure 2; Table A2 in Appendix 1). However, the proportion of female prey deliveries significantly increased with nestling age. The overall proportion of female prey deliveries did not significantly differ between wet and dry years or between warm and cold years.

### 3.3 Mass

**FIGURE 3:**
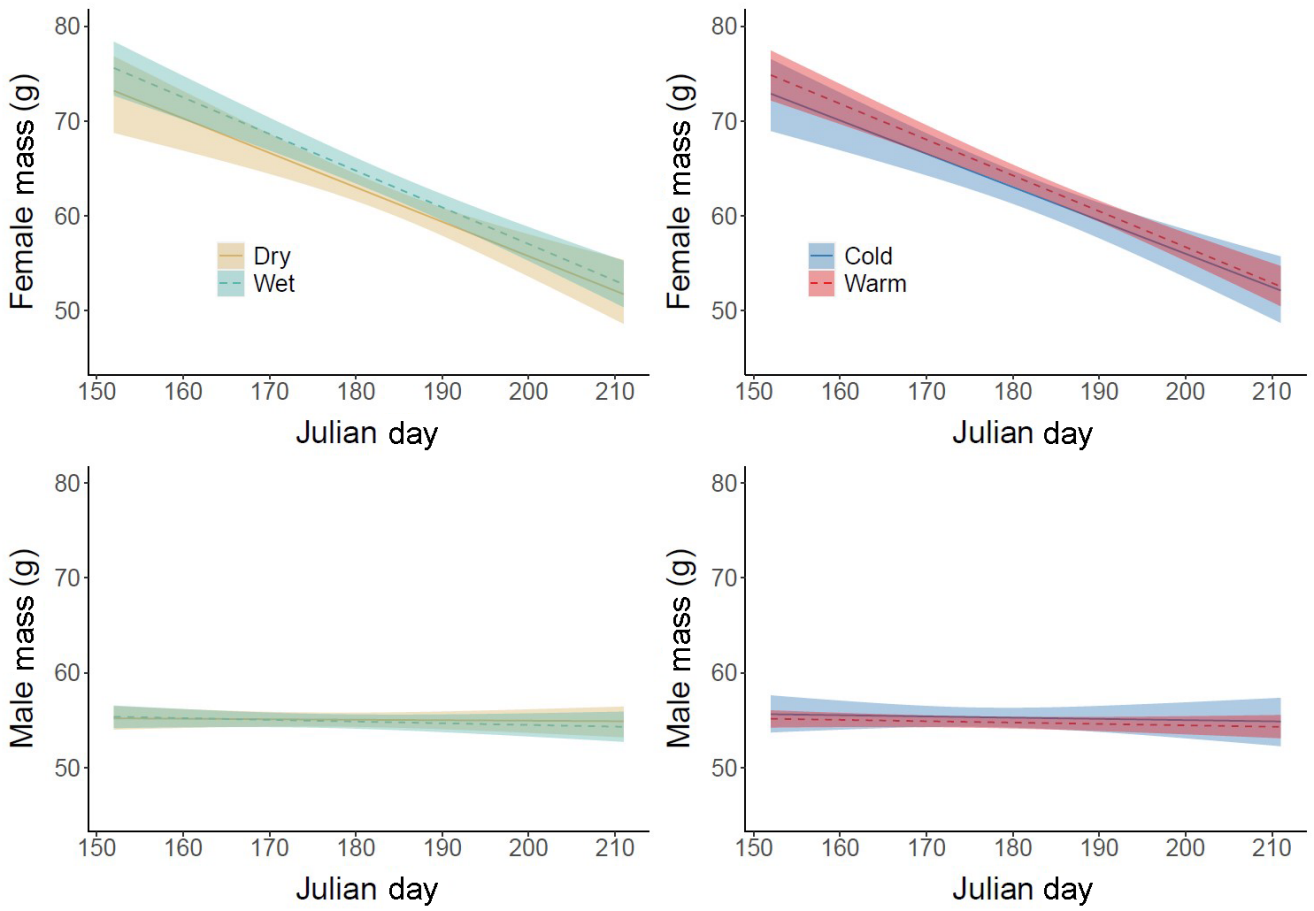
Bayesian linear mixed effects model (gaussian distribution) of adult female (top) and male (bottom) mass (g) throughout the nestling period. Solid and dashed lines represent the fitted value computed using the mean of posteriors, and shaded ribbons represent the 95% credible interval of posteriors. *Wet* = green/solid, *dry* = yellow/dashed, *warm* = red/dashed, *cold* = blue/solid.

We recorded the mass of 229 males and 174 females throughout the study period, including 77 females (44%) and 97 females (56%) in wet versus dry years, respectively; 95 females (55%) and 79 females (45%) in warm versus cold years; 102 males (45%) and 127 males (55%) in wet versus dry years; and 119 males (52%) and 110 males (48%) in warm versus cold years. We did not detect a significant difference in adult mass between wet and dry or between warm and cold years (Figure 3; Table A3 in Appendix 1). Female mass decreased significantly throughout the nestling period in all years, but male mass remained constant.

**FIGURE 4:**
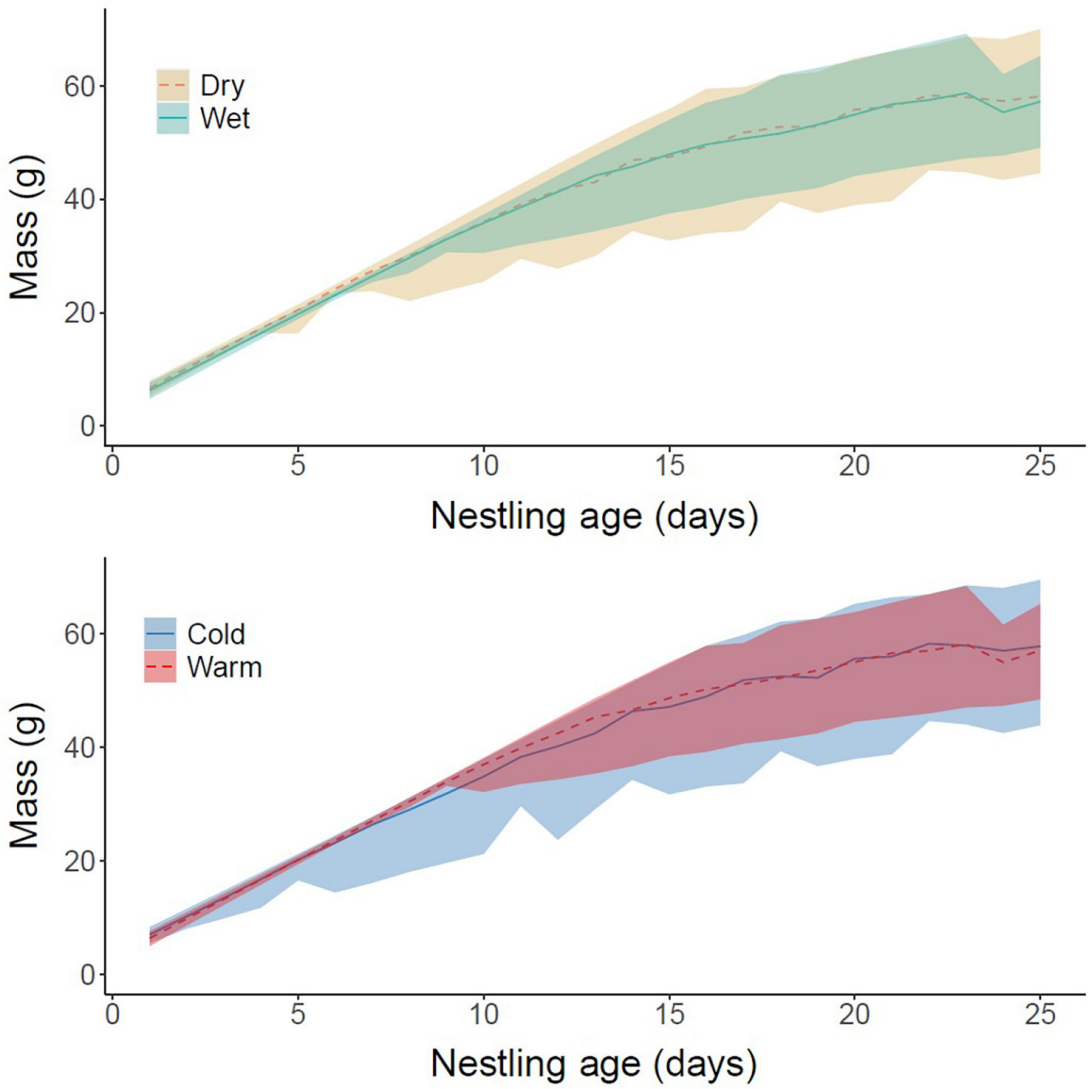
Bayesian changepoint model with random effects (gaussian distribution) of owlet mass (g) throughout the nestling period. Solid and dashed lines represent the fitted value computed using the mean of posteriors, and shaded ribbons represent the 95% credible interval of posteriors. *Wet* = green/solid, *dry* = yellow/dashed, *warm* = red/dashed, *cold* = blue/solid.

We recorded the mass of 108 nestlings throughout the study period, including 45 nestlings (42%) and 63 nestlings (58%) in wet versus dry years, respectively, and 54 nestlings (50%) and 54 nestlings (40%) in warm versus cold years. On average, each nestling was weighed 10.2 times (range = 2–27). Nestling growth rates also did not differ based on weather. All models showed evidence for a single changepoint that occurred around day 16 in wet, dry, warm, and cold years (Figure 4; Table A4 in Appendix 1). Slope estimates before and after the changepoint were not significantly different between wet and dry years or between warm and cold years. Intercept estimates were also highly overlapping, indicating similar owlet masses at hatching.

### 3.4 Productivity

**FIGURE 5:**
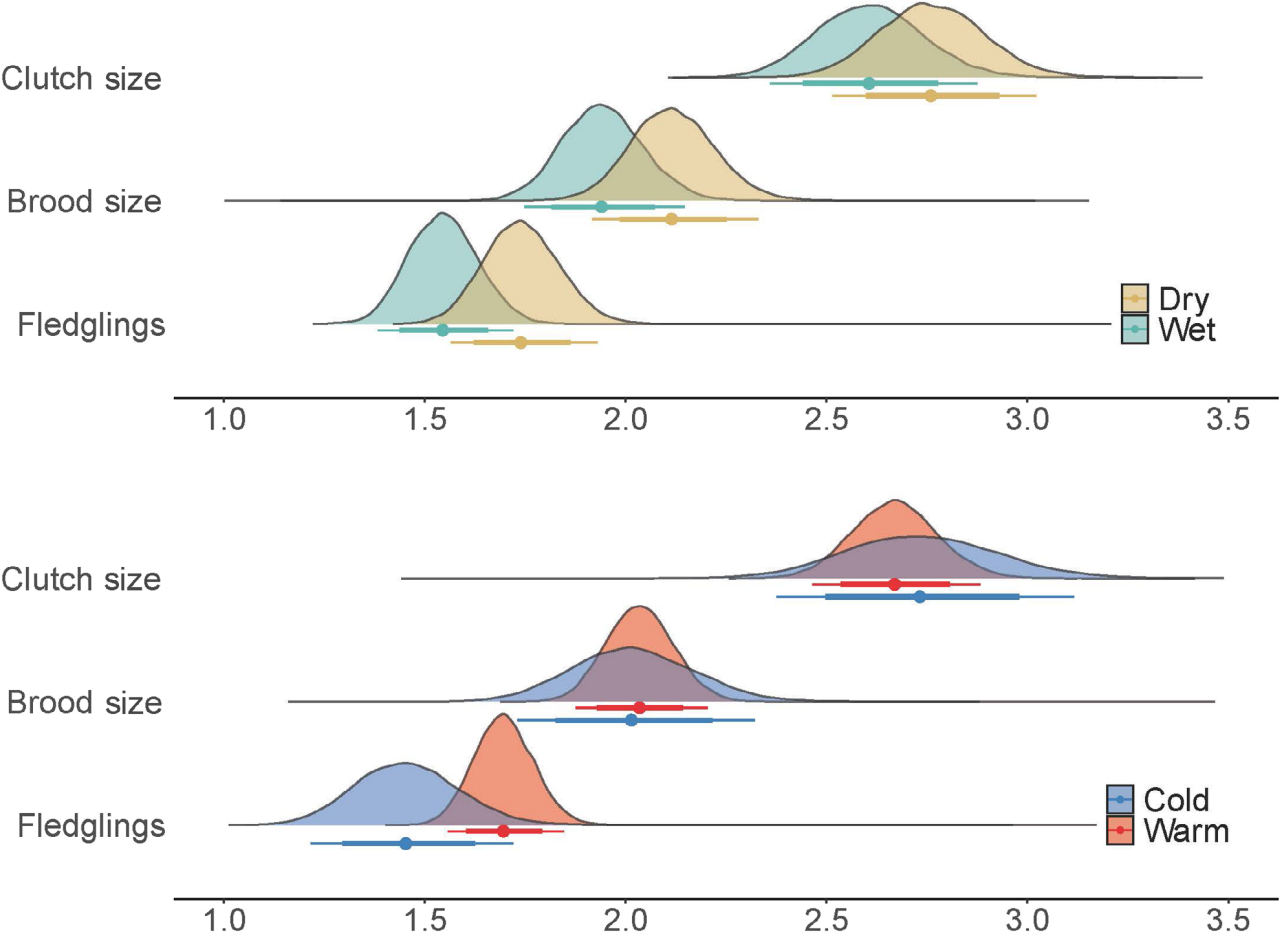
Posterior distribution of clutch size, brood size, and number of fledglings between *wet* and *dry* years (top) and between *warm* and *cold* years (bottom). Bars under the density plots represent the mean (dot), 80% credible interval (thick bar), and 95% credible interval (thin bar). *Wet* = green, *dry* = yellow, *warm* = red, *cold* = blue.

We did not detect a significant effect of *precipitation* on productivity. *Warm* years had a significant positive effect on the number of fledglings (*n* = 401 nests), but not on clutch (*n* = 319 nests) or brood (*n* = 381 nests) size (Table 4, Figure 5). All CRIs of the posterior mean overlapped between wet and dry years and between warm and cold years.

**TABLE 4:** Mean and 95% credible interval (CRI) for the posterior estimates of slope coefficients for nest productivity.

	Posterior slope estimate		
	Mean	Lower 2.5%	Upper 97.5%
Effect of wet years			
Clutch	−0.06	−0.10	0.08
Brood	−0.09	−0.13	0.05
Fledgling	−0.12	−0.17	0.04
Effect of warm years			
Clutch	−0.02	−0.07	0.14
Brood	0.01	−0.04	0.18
Fledgling*	**0.16**	**0.10**	**0.35**

*Note*: Values that do not overlap 0 (bold and marked with an asterisk) indicate a significant effect.

## 4 DISCUSSION

We found that the prey delivery rates were significantly higher in dry and cold years than in wet and warm years, both throughout a single night and throughout the nestling period. Further, prey deliveries increased at higher rates during the first part of the night and during the first part of the nestling period in dry and cold years. Taken together, these data suggest that in dry and cold years, either nestling energy demands were higher, high‐quality prey were less abundant, or both. Several previous studies have reported negative effects of drought (as reviewed by Barnett & Facey, 2016) and cold temperatures (Frampton et al., 2000; Grüebler et al., 2008) on insect abundance. Therefore, it is unlikely that more frequent prey deliveries are the result of more abundant prey. Preliminary blacklight data from our study area showed consistently higher abundances of micro‐Lepidoptera (families Depressariidae, Pyralidae, and Tortricidae) compared to medium‐bodied Lepidoptera (families Noctuidae and Geometridae; BDL, unpublished data), regardless of temperature or precipitation. If insect prey of all sizes are less abundant in dry and cold years, as previous studies have found, adults may compensate for the lack of large prey by making more frequent deliveries of the more abundant small, or low‐quality, prey (Cauchard et al., 2021; Mägi et al., 2009; McClenaghan et al., 2019).

Prey delivery rates dropped off much earlier in the night and earlier in the season in dry and cold years compared to wet and warm years. We hypothesize that earlier nightly changepoints are due to adult fatigue in dry and cold years, resulting from high effort early in the night. Earlier seasonal changepoints may be explained by high rates of prey deliveries taking a toll on adult health during the early nestling stage, thus inhibiting adults' ability to keep up these high rates late in the nestling stage. Differences in changepoints are not an apparent consequence of nestlings growing more rapidly in dry and cold years than in wet and warm years because models did not detect any difference in owlet growth rates based on weather. We do not expect that lengthening the duration of prey delivery observation beyond 90 min would lead to different conclusions because our models showed delivery rates dropping off well before 60 min postsunset. We do acknowledge, however, that predawn prey deliveries are also important for this species and were not evaluated in this study.

Later in the nestling period, prey delivery rates began to decrease in wet and warm years, while they continued to increase in dry and cold years. This elevated provisioning rate in cold years may indicate that late‐stage nestlings need more energy to maintain homeothermy than in warm years, which coincides with when females are no longer brooding and can increase prey delivery rates. Despite the fact that tree cavities provide some insulation from temperature fluctuations, studies have shown that internal cavity temperatures decline significantly when the ambient air temperature drops below 16°C (Vierling et al., 2018), which is a common nightly occurrence at our high‐elevation study site during the breeding season.

Precipitation in previous seasons can indirectly increase summer insect abundances by promoting plant growth, thus increasing food biomass for herbivorous insects and habitat for many other insects (Fay et al., 2003; Wu et al., 2011). The relationship between temperature and nest‐provisioning rates is not clearly delimited, with some studies showing positive correlations between temperature and nest visitations (Brown, 1976; Finlay, 1976; Low et al., 2008) and others, including our study, showing the opposite (Barras et al., 2021; Grüebler et al., 2008; Schifferli et al., 2014). Since our study is unique in its focus on a single‐prey loading species, further study is needed to determine the extent to which energetic costs of nest provisioning in extreme temperatures are mediated by the extent of prey loading. Further study is also needed to determine effects of daily and seasonal temperature changes on nest‐provisioning rates, thereby expanding on our focus on the full annual cycle to elucidate patterns over multiple temporal scales.

Adults worked harder in dry and cold years to deliver prey at higher rates, but the impact of this increased energy expenditure did not affect adult masses throughout the nestling period. Female mass decreased throughout the season, presumably because mass accumulated during laying and incubation was lost as females spent more time provisioning for the nest. Male mass, on the other hand, remained constant throughout the nestling period. These patterns in adult mass are common in other avian species that rely on a female‐only incubation strategy (Cichon et al., 1999; Durant et al., 2004; Moreno, 1989). While our findings indicate adults did not incur short‐term consequences of increased nest provisioning in dry and cold years, it is possible that Flammulated Owls will experience decreased adult survival if dry and cold years become more common. Given the importance of adult survival in maintaining stable population growth, particularly in birds with low reproductive rates like Flammulated Owls (Clark & Martin, 2007; Ludwig et al., 2018), future studies should determine effects of extreme weather on adult survival in insectivorous birds.

The effects of precipitation and temperature on prey delivery rates did not appear to manifest in nestling growth. Our findings are consistent with many other studies of the effects of weather on avian nestling development (Dyrcz & Czyż, 2018; Gullett et al., 2015; Kruuk et al., 2015), but some effects on nestling development have been detected in cases where climate differences were extreme (Pérez et al., 2016) or masses were measured immediately after intense bouts of rainfall (Cox et al., 2019).

Beyond a positive effect of warm years on the number of fledglings, we found that weather did not significantly affect productivity. The effects of precipitation and temperature on avian productivity varies in the literature, with most studies detecting no effect of these climate variables (Demay & Walters, 2019; Desante & Saracco, 2021; Gullett et al., 2015). However, some studies noted negative effects of heavy rainfall and low yearly temperatures on fledgling success (Ahola et al., 2009; Fisher et al., 2015). Unlike adult survival, nest success does not appear to be an important factor in determining population growth rates in species with small clutch sizes (Clark & Martin, 2007).

## 5 CONCLUSION

Yearly differences in precipitation and temperature have meaningful effects on the behavior of Flammulated Owls in a temperate forest ecosystem. While we cannot say whether wet, dry, warm, or cold years provide better breeding or foraging conditions for Flammulated Owls, it is clear that the climate in the year leading up to the breeding season impacts this species' breeding ecology. In the 17 years examined in our study, adult Flammulated Owls appeared to successfully compensate for yearly differences in precipitation and temperature by increasing nestling provisioning rates, thus offsetting the effects of climate on owlet growth and nest productivity. However, as global temperature and precipitation fluctuations intensify, we predict that the patterns identified in our study will become more pronounced and could result in effects on adult and nestling body condition, survival, and productivity in this and other populations. Flammulated Owls may be more insulated from climate fluctuations than other temperate species because of their relatively small clutch sizes, which show very little variability. Many other organisms have likely already begun to change their behaviors in response to increasing climate fluctuations, which will take a toll on many species' survival as patterns become more extreme. We recommend that future research investigate whether species of different guilds also exhibit changes in breeding ecology in response to year‐round weather. Additionally, determining whether short‐term sacrifices (e.g., increasing prey delivery rates) come at the expense of individual fitness will be an important step for assessing whether the responses elucidated in our study have meaningful, long‐term impacts on population growth.

## AUTHOR CONTRIBUTIONS

**Eliza D. Stein:** Conceptualization (supporting); formal analysis (lead); investigation (supporting); methodology (equal); visualization (lead); writing – original draft (lead); writing – review and editing (equal). **Stephen R. Midway:** Conceptualization (supporting); formal analysis (supporting); methodology (equal); visualization (supporting); writing – review and editing (equal). **Brian D. Linkhart:** Conceptualization (lead); formal analysis (supporting); funding acquisition (lead); investigation (lead); methodology (equal); project administration (lead); supervision (lead); visualization (supporting); writing – review and editing (equal).

## CONFLICT OF INTEREST STATEMENT

The authors do not have any conflict of interest relevant to this project.

### OPEN RESEARCH BADGES

This article has earned an Open Data badge for making publicly available the digitally‐shareable data necessary to reproduce the reported results. The data is available at https://doi.org/10.6084/m9.figshare.22277725 and https://doi.org/10.6084/m9.figshare.22277731.

## Data Availability

Prey delivery observations, adult and owlet masses, productivity data, and R scripts for all analyses are publicly available on FigShare at https://doi.org/10.6084/m9.figshare.22277725 and https://doi.org/10.6084/m9.figshare.22277731.

## APPENDIX 1

**TABLE A1:** Akaike Information Criterion (AIC) values for Fisherian models tested before conducting Bayesian analysis.

Model	AIC
**Annual (June–May) total monthly precipitation**	**7952**
June–July total precipitation	7980
January–June total precipitation	7981
July–December total precipitation	7978
Annual (June–May) mean daily temperature	7948
Annual (June–May) mean of maximum daily temperature	7977
**Annual (June–May) mean of minimum daily temperature**	**7919**
June–July mean daily temperature	7969
June–July mean of maximum daily temperature	7964
June–July mean of minimum daily temperature	7961

*Note*: Parameters chosen for inclusion in the final Bayesian models are shown in bold.

**TABLE A2:** Mean and 95% credible interval (CRI) of posterior estimates of the slope coefficient for the proportion of prey deliveries given by the female.

	Posterior slope estimate		
	Mean	Upper 97.5	Lower 2.5
Time after sunset			
Wet	6.35E‐03	1.45E‐02	−1.99E‐03
Dry	−3.53E‐03	1.52E‐03	−8.55E‐03
Warm	2.30E‐03	8.33E‐03	−3.73E‐03
Cold	−4.35E‐03	2.28E‐03	−1.10E‐02
Nestling age			
*Wet*	*5.48E‐02*	*7.80E‐02*	*3.14E‐02*
*Dry*	*4.91E‐02*	*6.44E‐02*	*3.33E‐02*
*Warm*	*4.36E‐02*	*6.07E‐02*	*2.72E‐02*
*Cold*	*6.25E‐02*	*8.17E‐02*	*4.31E‐02*

*Note*: Values that do not overlap 0 (italicized) indicate a significant change over time. All CRIs overlap between *wet*/*dry* and *warm*/*cold* years, suggesting no difference based on climate.

**TABLE A3:** Mean and 95% credible interval (CRI) of posterior estimates of the slope coefficient for the change in adult mass over the nestling period.

	Posterior slope estimate		
	Mean	Upper 97.5	Lower 2.5
Female			
*Wet*	*−3.87E‐01*	*−3.03E‐01*	*−4.62E‐01*
*Dry*	*−3.63E‐01*	*−2.28E‐01*	*−4.65E‐01*
*Warm*	*−3.78E‐01*	*−3.05E‐01*	*−4.48E‐01*
*Cold*	*−3.51E‐01*	*−2.37E‐01*	*−4.55E‐01*
Male			
Wet	−1.80E‐02	2.24E‐02	−5.69E‐02
Dry	−5.20E‐03	3.30E‐02	−4.70E‐02
Warm	−1.45E‐02	1.75E‐02	−4.34E‐02
Cold	−1.29E‐02	4.91E‐02	−8.55E‐02

*Note*: Values that do not overlap 0 (italicized) indicate a significant change over time. All CRIs overlap between *wet*/*dry* and *warm*/*cold* years, suggesting no difference based on climate.

**TABLE A4:** Mean and 95% credible interval (CRI) of posterior estimates of changepoints, slopes, and intercepts for owlet mass changepoint models.

	Posterior estimate		
	Mean	Upper 97.5	Lower 2.5
Changepoint			
Wet	16.49	15.00	18.16
Dry	16.11	13.34	18.00
Warm	15.86	15.00	16.97
Cold	17.23	13.08	19.00
Slope 1			
Wet	2.84	2.66	3.01
Dry	2.88	2.72	3.06
Warm	2.95	2.80	3.10
Cold	2.74	2.57	2.92
Slope 2			
Wet	0.47	0.01	0.83
Dry	0.40	−0.03	0.82
Warm	0.33	0.04	0.61
Cold	0.48	−0.10	1.15
Intercept			
Wet	6.37	4.56	8.19
Dry	6.23	4.60	7.82
Warm	6.26	4.78	7.74
Cold	6.12	4.36	7.95

*Note*: All CRIs for changepoint, slopes, and intercept overlap between *wet*/*dry* and *warm*/*cold* years, suggesting no difference based on climate.
